# Genome-wide prediction of disease variant effects with a deep protein language model

**DOI:** 10.1038/s41588-023-01465-0

**Published:** 2023-08-10

**Authors:** Nadav Brandes, Grant Goldman, Charlotte H. Wang, Chun Jimmie Ye, Vasilis Ntranos

**Affiliations:** 1 Division of Rheumatology, Department of Medicine, University of California, San Francisco, San Francisco, CA USA; 2 Biological and Medical Informatics Graduate Program, University of California, San Francisco, San Francisco, CA USA; 3 Biomedical Sciences Graduate Program, University of California, San Francisco, San Francisco, CA USA; 4 Bakar Computational Health Sciences Institute, University of California, San Francisco, San Francisco, CA USA; 5 Parker Institute for Cancer Immunotherapy, University of California, San Francisco, San Francisco, CA USA; 6 Gladstone-UCSF Institute of Genomic Immunology, San Francisco, CA USA; 7 Institute for Human Genetics, University of California, San Francisco, San Francisco, CA USA; 8 Department of Epidemiology & Biostatistics, University of California, San Francisco, San Francisco, CA USA; 9 Department of Bioengineering and Therapeutic Sciences, University of California, San Francisco, San Francisco, CA USA; 10 Diabetes Center, University of California, San Francisco, San Francisco, CA USA

**Keywords:** Functional genomics, Bioinformatics

## Abstract

Predicting the effects of coding variants is a major challenge. While recent deep-learning models have improved variant effect prediction accuracy, they cannot analyze all coding variants due to dependency on close homologs or software limitations. Here we developed a workflow using ESM1b, a 650-million-parameter protein language model, to predict all ~450 million possible missense variant effects in the human genome, and made all predictions available on a web portal. ESM1b outperformed existing methods in classifying ~150,000 ClinVar/HGMD missense variants as pathogenic or benign and predicting measurements across 28 deep mutational scan datasets. We further annotated ~2 million variants as damaging only in specific protein isoforms, demonstrating the importance of considering all isoforms when predicting variant effects. Our approach also generalizes to more complex coding variants such as in-frame indels and stop-gains. Together, these results establish protein language models as an effective, accurate and general approach to predicting variant effects.

## Abstract (web summary)

A modified framework leveraging a protein language model (ESM1b) is used to predict all possible 450 million missense variant effects in the human genome and shows potential for generalizing to more complex genetic variations such as indels and stop-gains.

## Main

Determining the phenotypic consequences of genetic variants, known as variant effect prediction (VEP), is a key challenge in human genetics^1–4^. Coding variants altering the amino acid sequences of proteins are of special interest due to their enrichment in disease associations, better-understood mechanisms and therapeutic actionability^5–8^. Most naturally occurring coding variants are missense, substituting one amino acid with another^9^. Despite progress in functional genomics and genetic studies, distinguishing protein-disrupting damaging variants from neutral ones remains a challenge. Furthermore, most human genes are alternatively spliced, and the same variant may be damaging to some protein isoforms but neutral to others, depending on interactions with the rest of the protein. Thus, most missense variants remain as variants of uncertain significance (VUS), limiting the utility of exome sequencing in clinical diagnosis^2,10^. VEP is even more challenging for coding variants affecting multiple residues such as in-frame indels.

Experimental approaches for VEP such as deep mutational scans (DMS)^11^ and Perturb-seq^12^ can measure molecular and cellular phenotypes across thousands of variants simultaneously. However, these endophenotypes are imperfect proxies for the relevant clinical phenotypes and remain difficult to scale genome-wide^13,14^. In contrast, computational methods that learn the biophysical properties or evolutionary constraints of proteins could theoretically cover all coding variants^15–17^. While most computational methods are trained on labeled data of pathogenic versus benign variants^10^, unsupervised homology-based methods predict variant effects directly from multiple sequence alignments (MSA) without training on labeled data. EVE, an unsupervised deep-learning method implementing a generative variational autoencoder, was recently shown to outperform supervised methods^4^. However, due to their reliance on MSA, homology-based methods provide predictions only for a subset of well-aligned proteins and residues. Moreover, because alternative isoforms of the same gene have identical homologs, it is unclear whether they can distinguish the effects of variants on different isoforms.

**Fig. 1:**
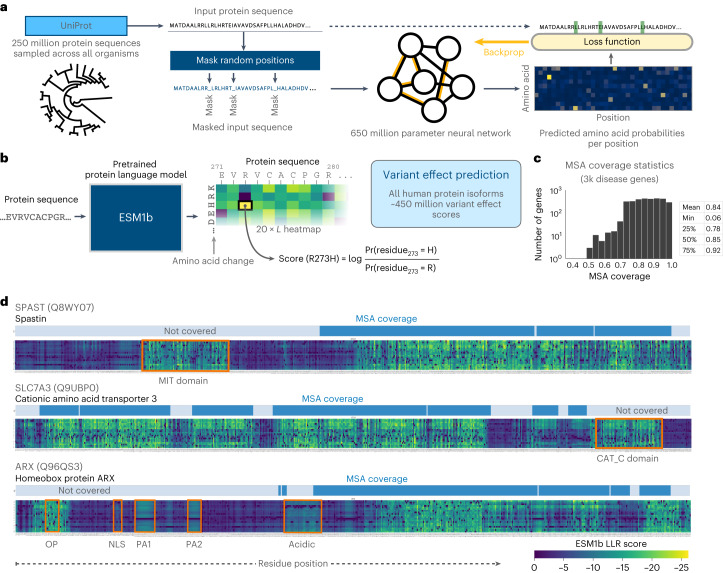
ESM1b predicts variant effects without homology coverage. **a**, ESM1b is a 650-million-parameter protein language model trained on 250 million protein sequences across all organisms. The model was trained via the masked language modeling task, where random residues are masked from input sequences and the model has to predict the correct amino acid at each position (including the missing residues). **b**, Illustration of the ESM1b model’s input (an amino acid sequence) and output (LLR of effect scores for all possible missense variants). **c**, The distribution of MSA coverage (that is, the fraction of a protein’s residues that are aligned) across ~3,000 disease-related proteins covered by EVE. **d**, Examples of the model’s capacity to detect protein domains and functional regions, including outside MSA coverage, across the following three human proteins: SPAST, SLC7A3 and ARX. Each heatmap visualizes the LLR scores across all 20 × *L* possible missense variants (where *L* is the protein length). Protein domains without MSA coverage are highlighted in orange.

Another deep-learning approach to VEP uses protein language models, a technique derived from natural language processing. These are deep neural networks trained to model the space of known protein sequences selected throughout evolution as captured by large protein datasets such as UniProt^18^ (Fig. 1a). Notably, protein language models do not require explicit homology and can estimate the likelihood of any possible amino acid sequence. They have been shown to implicitly learn how protein sequence determines many aspects of protein structure and function, including secondary structure, long-distance interactions, post-translational modifications and binding sites^19–24^. One of the largest protein language models is ESM1b, a publicly available 650-million-parameter model trained on ~250 million protein sequences^20^. It was demonstrated to predict, without further training, variant effects correlated with DMS experiment results^25^.

However, several limitations have restricted the use of ESM1b for VEP. First, the model’s input sequence length is limited to 1,022 amino acids, excluding ~12% of human protein isoforms. Second, while evaluated on DMS data across 32 genes (10 from humans)^25^, it has remained unknown how the model performs at predicting the clinical impact of coding variants genome-wide. Finally, using ESM1b requires software engineering proficiency, deep-learning expertise and high-memory GPUs, which together create a technical barrier for widespread use.

Here we implemented a workflow generalizing ESM1b to protein sequences of any length and used it to predict all ~450 million possible missense variant effects across all 42,336 protein isoforms in the human genome. We evaluated our workflow on three different benchmarks and compared it to 45 other VEP methods. Our workflow outperforms all compared methods in classifying variant pathogenicity (as annotated by ClinVar^10^ and HGMD^26^) and predicting DMS experiments. We further demonstrate the capacity of ESM1b to assess variant effects in the context of different protein isoforms, identifying isoform-sensitive variants in 85% of alternatively spliced genes. Finally, we present a scoring algorithm that generalizes ESM1b to variants affecting multiple residues and demonstrates the model’s accurate predictions over in-frame indels and stop-gain variants. We created a web portal allowing users to query, visualize and download missense VEPs for all human protein isoforms (accessible at https://huggingface.co/spaces/ntranoslab/esm_variants).

## Results

### Predicting the effects of all possible missense variants in the human genome

We developed a modified ESM1b workflow and applied it to obtain a complete catalog of all ~450 million missense variant effects on all 42,336 known human protein isoforms. Each variant’s effect score is the log-likelihood ratio (LLR) between the variant and wild-type (WT) residue (Fig. 1b). Unlike homology-based models currently available only for a subset of human proteins and residues with MSA coverage (for example, 84% of the residues in ~3,000 disease genes covered by EVE; Fig. 1c), ESM1b predicts the effects of every possible missense variant.

Protein regions with many possible mutations predicted by ESM1b as damaging often align with known protein domains (Fig. 1d). As illustrated for *SPAST*, *SLC7A3* and *ARX*, these domains may reside outside MSA coverage and be unsuitable for homology-based models (Fig. 1d), yet harbor disease-associated variants. For example, the microtubule-interacting and trafficking (MIT) domain in *SPAST* contains missense variants implicated in hereditary spastic paraplegias^27^, the CAT C domain in *SLC7A3* contains an autism-linked variant (S589T)^28^ and multiple domains in *ARX* outside MSA coverage (highlighted in Fig. 1d) contain missense variants linked to intellectual disability^29–32^.

### ESM1b outperforms other VEP methods over clinical and experimental benchmarks

**Fig. 2:**
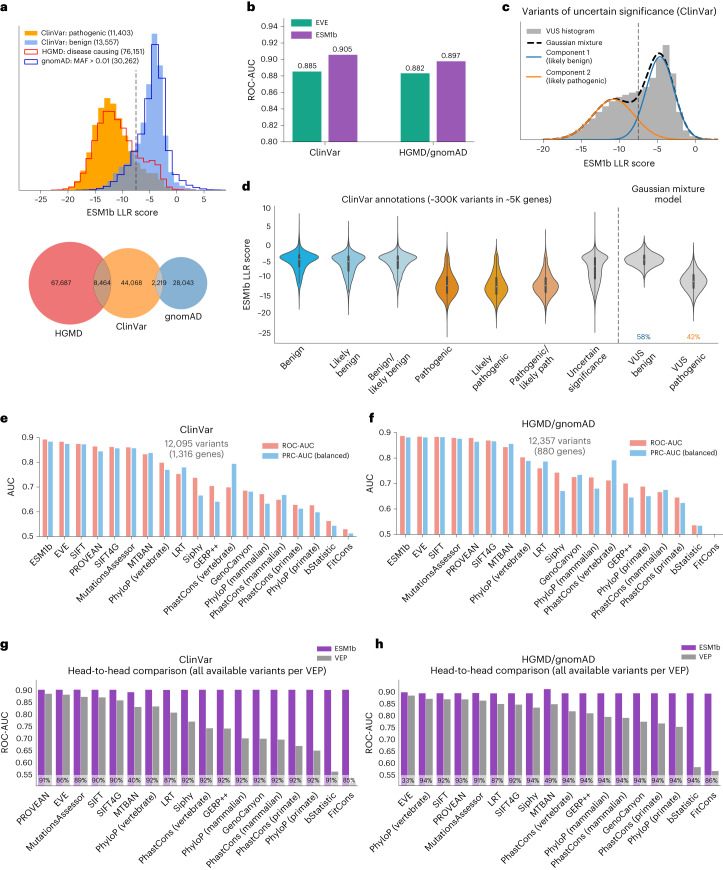
ESM1b is suitable for genome-wide disease prediction of coding variants. **a**, Top: the distribution of ESM1b effect scores across two sets of variants that are assumed to be mostly pathogenic (‘ClinVar: pathogenic’ and ‘HGMD: disease causing’) and two sets of variants assumed to be mostly benign (‘ClinVar: benign’ and ‘gnomAD: MAF > 0.01’). Bottom: Venn diagram of the variants extracted from HGMD, ClinVar and gnomAD. **b**, Comparison between ESM1b and EVE in their capacity to distinguish between pathogenic and benign variants (measured by global ROC-AUC scores), as labeled by ClinVar (36,537 variants in 2,765 unique genes) or HGMD/gnomAD (30,497 variants in 1,991 unique genes). **c**, The distribution of ESM1b effect scores across ClinVar missense VUS, decomposed as a mixture of two Gaussian distributions capturing variants predicted as more likely pathogenic (orange) or more likely benign (blue). **d**, The distribution of ESM1b effect scores across all common ClinVar labels, including the two Gaussian components from **c**. Boxes mark Q1–Q3 of the distributions, with midpoints marking the medians (Q2) and whiskers stretching 1.5× IQR. Altogether there are ~300,000 missense variants labeled in ClinVar. **e**,**f**, Evaluation of 19 VEP methods against the same two benchmarks: ClinVar (**e**) and HGMD/gnomAD (**f**). Performance was measured by two metrics for binary classification as follows: ROC-AUC (light red) and a balanced version of PRC-AUC (light blue; Methods). Performance was evaluated on the sets of variants available for all 19 methods. **g**,**h**, Head-to-head comparison between ESM1b and each of the 18 other VEP methods over the same two dataset benchmarks (in terms of ROC-AUC). Because ESM1b provides scores for all missense mutations, the comparison against each other method is performed on the set of variants with effect predictions for that method. The percentage of variants considered for each method is shown at the bottom of each bar. IQR, interquartile range.

To assess the performance of ESM1b in predicting the clinical impact of variants, we compared the model’s effect scores between pathogenic and benign variants in two datasets. The first dataset contains pathogenic and benign variants annotated in ClinVar^10^ and the second includes variants annotated by HGMD as disease-causing^26^ and benign variants from gnomAD (defined by allele frequency >1%)^9^. Only high-confidence variants were included (Supplementary Methods). The distribution of ESM1b effect scores shows a substantial difference between pathogenic and benign variants in both datasets (Fig. 2a). Moreover, pathogenic and benign variants show consistent distributions across the two datasets, suggesting that the predictions are well-calibrated. Using an LLR threshold of −7.5 to distinguish between pathogenic and benign variants yields a true-positive rate of 81% and a true-negative rate of 82% in both datasets.

**Extended Data Fig. 1:**
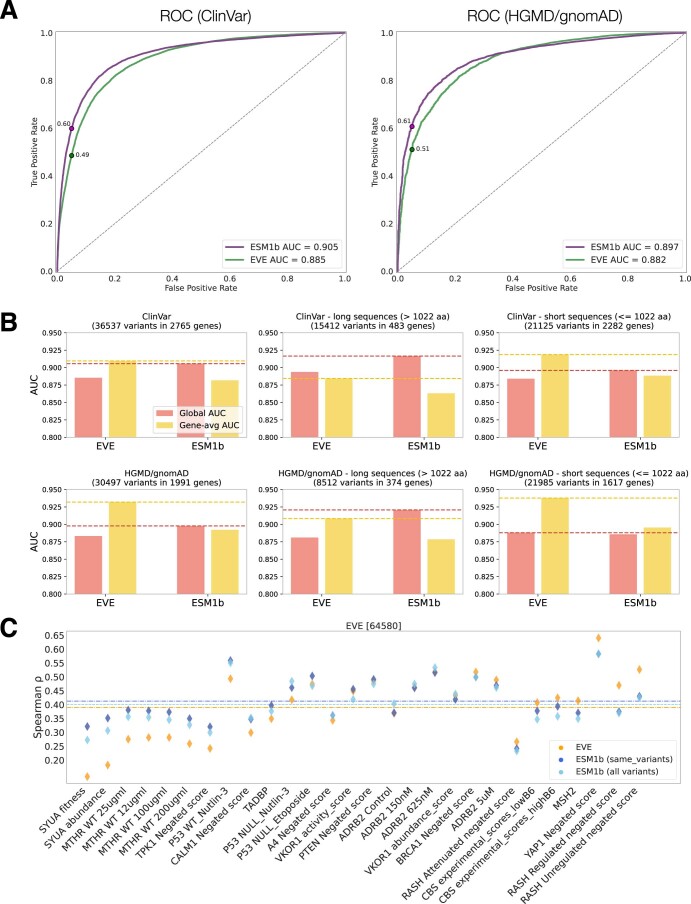
Comprehensive evaluation of ESM1b and EVE on ClinVar, HGMD/gnomAD and deep mutation scans. (**a**) ROC curves of ESM1b and EVE as binary classifiers of variant pathogenicity over ClinVar (left) and HGMD/gnomAD (right). The true positive rate at the standard false positive rate (0.05) is annotated across all 4 curves. (**b**) Evaluation of EVE (left bar plots) and ESM1b (right bar plots) over ClinVar (top panels) and HGMD/gnomAD (bottom panels), using either the global ROC-AUC (red) or gene-average ROC-AUC (yellow) metric (see the relevant section in the Methods). For each dataset, we show the results for either the full dataset (left panels), or the subsets of variants in long (middle panels) or short (right panels) proteins (defined by a threshold of 1,022aa, which is the maximum window length supported by ESM1b; see Methods). Dashed lines: the top score (obtained by ESM1b or EVE) according to each of the two metrics. (**c**) Evaluation of ESM1b and EVE on deep mutational scanning datasets over each of the 28 assays (which were aggregated per gene in Fig. 3b).

Comparing ESM1b and EVE as classifiers of variant pathogenicity, ESM1b obtains a receiver operating characteristics–area under the curve (ROC-AUC) score of 0.905 for distinguishing between the 19,925 pathogenic and 16,612 benign variants in ClinVar (across 2,765 genes), compared to 0.885 for EVE. On HGMD/gnomAD (with 27,754 disease-causing and 2,743 common variants across 1,991 genes), ESM1b obtains a ROC-AUC score of 0.897 compared to 0.882 for EVE (Fig. 2b). We also considered a gene-specific ROC-AUC metric, where ESM1b performs slightly worse. However, we consider the global metric more suited for genome-wide scanning of disease variants, where comparing variants across different genes is often necessary (Extended Data Fig. 1b and Methods).

The ROC curve shows the true-positive rate (percentage of pathogenic variants successfully predicted as such) for every possible false-positive rate (of benign variants mistakenly predicted pathogenic). While the ROC-AUC metric assesses overall model performance by integrating overall false- and true-positive rates, clinical applications usually require low false-positive rates. At a false-positive rate of 5%, ESM1b obtains a 60% true-positive rate compared to 49% for EVE over ClinVar and 61% compared to 51% over HGMD/gnomAD (Extended Data Fig. 1a), showing a substantial margin at the clinically relevant regime of the ROC curve.

Having established the high accuracy of ESM1b as a classifier of variant pathogenicity, we sought to predict the effects of VUS in ClinVar. To that end, we modeled the distribution of ESM1b effect scores across VUS as a Gaussian mixture with two components (Fig. 2c). These two fitted distributions align well with the distributions for annotated pathogenic and benign variants (Fig. 2d). According to this model, we estimate that about 58% of missense VUS in ClinVar are benign and about 42% are pathogenic.

In addition to EVE, we compared ESM1b to 44 other VEP methods, including all functional prediction methods and conservation scores from the Database for Nonsynonymous SNPs’ Functional Predictions (dbNSFP)^33^. For clinical benchmark comparisons, we only considered methods that (1) were not trained on clinical databases such as ClinVar and HGMD or used features from methods trained on such data, and (2) do not use allele frequency as a feature, as it is often used to curate variants as benign. Of the 46 methods, 19 (including ESM1b and EVE) satisfy these criteria for an unbiased comparison. Over the set of variants reported by all 19 methods, ESM1b outperforms all other methods on both ClinVar and HGMD/gnomAD (Fig. 2e,f). Similarly, ESM1b outperforms each method separately on its respective set of reported variants (Fig. 2g,h). All head-to-head comparisons were statistically significant with *P* < 0.001. Evaluation results for all 46 methods, including those excluded for data leakage concerns, are reported in Supplementary Table 2.

**Fig. 3:**
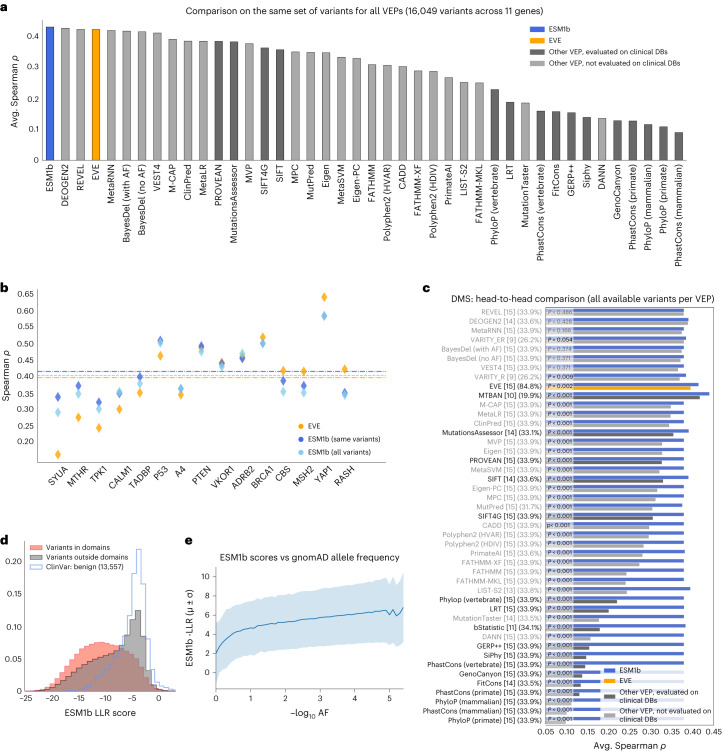
ESM1b predicts the effects of experimental measurements from DMS. **a**, Evaluation of 43 VEP methods (including ESM1b and EVE) on a DMS benchmark containing 28 assays over 15 different human genes (Supplementary Table 1). Of the entire set of 76,133 variants in 15 genes, 16,049 variants in 11 genes obtained effect scores by all 43 VEP methods. We excluded 3 VEP methods, VARITY_ER, VARITY_R and MTBAN (Methods), which would have dramatically reduced the number of variants and genes shared by all methods. The methods are sorted by the average Spearman’s correlation between each method’s scores and the experimental scores. **b**, The performance of ESM1b and EVE over the 15 individual genes in the DMS benchmark. The average performance of each method is marked by a dashed line. Because ESM1b can process all missense variants (while EVE assigns scores only for a subset of them), the performance of ESM1b is shown either for all variants (‘all variants’) or the subset of variants with EVE scores (‘same variants’). **c**, Head-to-head comparison between ESM1b and each of the other 45 VEP methods on the DMS benchmark, where each method is compared against the set of variants with predictions for that method. The number of unique genes and percentage of variants with predictions for each method are shown in squared brackets and parentheses, respectively. One-tailed *P* values indicating significant differences from ESM1b are shown at the beginning (left) of the bars. Methods are sorted by the difference in average Spearman’s correlation between ESM1b and each of the other methods. Comparisons against methods not evaluated on clinical DBs are grayed out. **d**, The distribution of ESM1b effect scores for variants in annotated protein domains (red) versus variants outside of domains (gray). The distribution of benign variants (as in Fig. 2a) is shown for reference. **e**, Average ESM1b effect score (and s.d.) as a function of allele frequency over all gnomAD missense variants.

**Extended Data Fig. 2:**
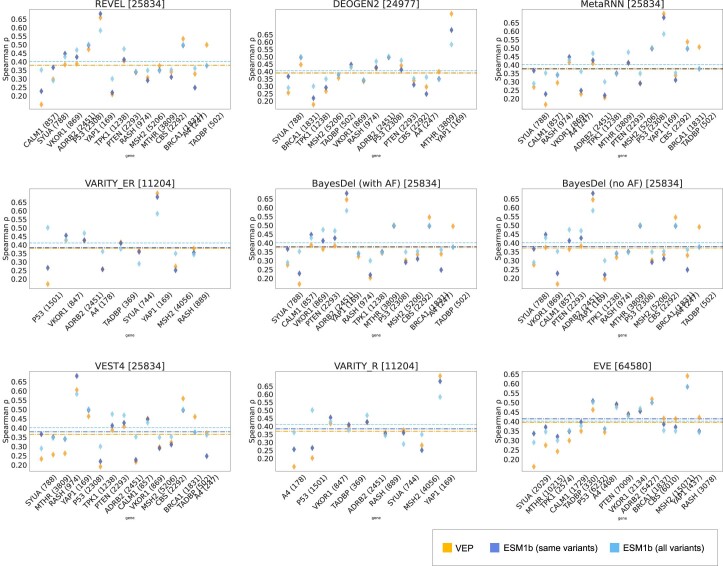
Per-gene evaluation of top VEP methods on deep mutational scans. Per-gene DMS results for the 9 VEP methods that are closest to ESM1b in performance according to the head-to-head comparison (Fig. 3c). The numbers of unique variants scored by each VEP method, out of the total 76,133 variants in the full DMS dataset, are shown in square brackets next to the method names. The numbers of variants per gene are shown in parentheses next to the gene names.

We further compared all 46 VEP methods in their ability to predict experimental measurements from DMS. The full DMS benchmark consists of 28 assays covering 15 human genes (166,132 experimental measurements over 76,133 variants; Supplementary Table 1). We compared 43 of the methods against a subset of 16,049 variants across 11 genes reported by these methods (excluding 3 methods that would have greatly reduced the number of shared variants; Methods). ESM1b is ranked highest with a mean Spearman’s correlation of 0.426 between its effect scores and the experimental measurements (Fig. 3a), followed by DEOGEN2 (0.423), REVEL (0.419) and EVE (0.418). DEOGEN2 and REVEL are supervised methods, whereas EVE, like ESM1b, is an unsupervised method. Comparing ESM1b and EVE head-to-head against the 64,580 variants with EVE scores (across 15 genes) shows a similar trend (Fig. 3b and Extended Data Fig. 1c). Likewise, ESM1b outperforms all 45 other methods over the set of variants reported by each method (Fig. 3c and Extended Data Fig. 2), with 37 of 45 comparisons statistically significant (*P* < 0.05).

Two additional analyses further demonstrate the functional interpretation of ESM1b predictions. First, as illustrated by individual examples (Fig. 1d), missense variants within domains have more negative (damaging) effect scores, while those outside domains resemble benign variants (Fig. 3d). Second, ESM1b effect scores track well with allele frequency, with common variants predicted less damaging (Fig. 3e), consistent with purifying selection eliminating highly deleterious mutations^34,35^.

### ESM1b can predict variant effects on alternative protein isoforms

**Fig. 4:**
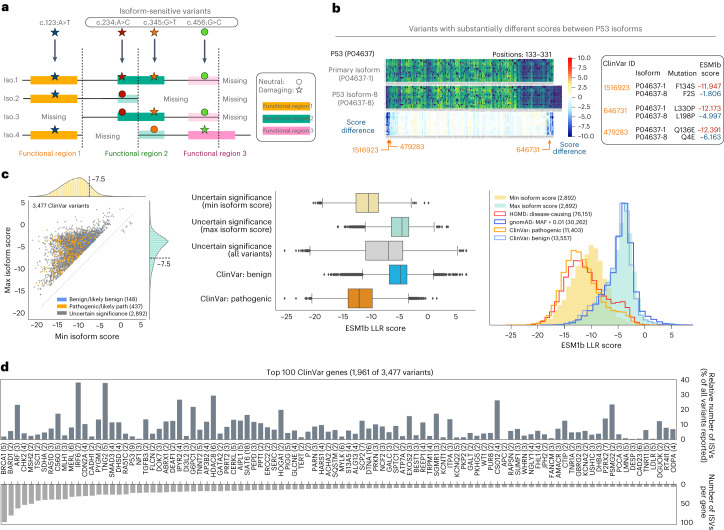
ESM1b predictions in clinically relevant genes depend on the isoform context. **a**, The consequences of variants (for example, damaging versus neutral) can depend on the isoform context. **b**, Comparison of the primary and one of the alternative isoforms of P53. Three specific variants are detailed. **c**, Left: all 3,477 ClinVar variants with highly variable ESM1b effect scores across different isoforms (defined by s.d. > 2). Center: the lowest and highest isoform scores predicted for all VUS from the left panel (top two boxes), compared to the mean scores (across isoforms) of VUS, benign or pathogenic variants (as in Fig. 2d; bottom three boxes). The boxes represent the Q1–Q3 range and median (Q2) line; whiskers correspond to 1.5× IQR; outliers (outside the whiskers) are shown individually. Right: the distribution of the lowest and highest isoform scores predicted for all VUS from the left panel, compared to the distributions for pathogenic or benign variants from ClinVar, HGMD and gnomAD (as in Fig. 2a). Across all panels, the number of variants associated with each category is shown in parentheses. **d**, The top 100 ClinVar genes with the highest number of variants with highly variable effect scores (as in **c**). Numbers of annotated isoforms of each gene are shown in parentheses.

As a protein language model, ESM1b assesses each variant in the context of the input amino acid sequence, allowing the same variant to be assessed in the context of different protein isoforms. A variant might be damaging to some isoforms but not others, possibly due to interactions with alternatively spliced domains (Fig. 4a). For example, comparing ESM1b scores between the primary and a shorter isoform of P53 (known as Δ133p53β)^36^, we found 170 variants (mostly near the splice junctions) with substantially different scores (LLR difference > 4), including three ClinVar variants annotated as VUS (Fig. 4b).

We found 3,477 missense variants in ClinVar with substantial differences in predicted effects (LLR s.d. > 2) across isoforms (Fig. 4c). Notably, we only considered reviewed, manually curated protein isoforms (Supplementary Methods). These 3,477 variants include 148 (4%) benign or likely benign, 437 (13%) pathogenic or likely pathogenic and 2,892 (83%) VUS. Interestingly, these VUS mirror the effect score distribution of pathogenic variants when considering the most damaging isoform, and benign variants when considering the least damaging isoform (Fig. 4c). Like P53, many clinically important genes have a large number of ClinVar variants with high effect score variance across isoforms, including *BRCA1*, *IRF6* and *TGFB3* (Fig. 4d).

**Fig. 5:**
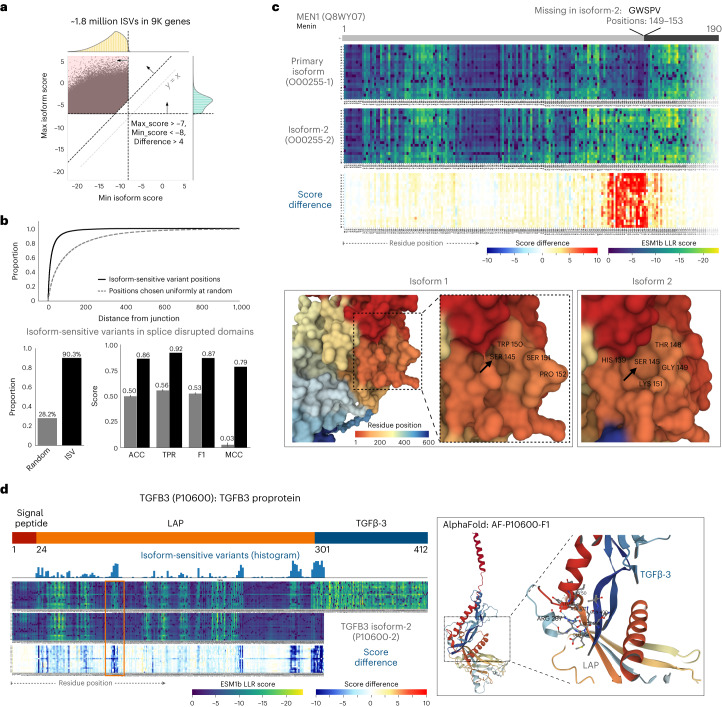
ESM1b can detect isoform-specific variant effects. **a**, Approximately 1.8 million missense variants across ~9,000 genes in the human genome are ‘isoform sensitive’, defined by (1) maximum ESM1b effect score (across isoforms) > −7, (2) minimum score < −8 and (3) difference between minimum and maximum score > 4. **b**, Top: ISV are closer to splice junction than would be expected at random. Bottom-left: ISV in genes with domains containing splice junctions: 90.31% versus 28.21% expected at random. Bottom-right: metrics of predicting whether genes contain domains disrupted by splice junction given whether or not they contain ISV. **c**, An example of a small splicing effect (excision of five amino acids from the primary isoform of the MEN1 protein) leading to dramatic changes in the predicted effects of variants in a much larger region. Bottom: AlphaFold structural predictions of the two isoforms. Arrows are pointing to a small surface pocket introduced by the five amino acid deletion (around Ser145). **d**, An example of alternative splicing leading to a distant effect in the TGFB3 proprotein. Exclusion of the TGFβ-3 chain in an alternative isoform of the proprotein leads to a region at the beginning of the LAP chain (marked by orange) losing its sensitivity to missense variants. Right: AlphaFold prediction of the binding of the two chains showing these two regions to be close to one another in 3D structure. ISV, isoform-sensitive variants; ACC, accuracy; TPR, true-positive rate; F1, F1 score; MCC, Matthew’s correlation coefficient.

Beyond the ~5,000 ClinVar genes, we searched for isoform-specific effects across all possible missense variants in all 20,360 coding human genes. We define a variant to be isoform-sensitive according to ESM1b if (1) it is likely benign (LLR > −7) in one isoform, (2) likely pathogenic (LLR < −8) in another and (3) these two predictions are substantially different (LLR difference > 4). We identified ~1.8 million such variants across ~9,000 genes, which is 85% of all genes with manually curated alternative isoforms (Fig. 5a). Isoform-sensitive variants (ISV) are more likely to occur near splice junctions and in genes with splicing-disrupted protein domains, as opposed to domains that are either included intact or removed entirely during splicing (Fig. 5b).

Splicing events can dramatically influence predicted variant effects. For example, the second isoform of MEN1, a tumor suppressor involved in many cancers, differs from the primary isoform by only five amino acids deleted at positions 149–153. Differences in predicted variant effects between the isoforms suggest that this small deletion introduces a 30 amino acid region that is more prone to damaging variants in the second MEN1 isoform (Fig. 5c). Multiple studies have associated missense variants in that region with cancer, suggesting that it may be functional^37–42^. A 2017 study found aberrant expression of the second MEN1 isoform in tumors, but the functional differences between the two isoforms remain uncharacterized^43^. Comparing predicted three-dimensional (3D) structures^44^, we observe a small surface pocket introduced by the five amino acid deletion (Fig. 5c), further supporting its functional relevance. However, caution is advised when using one computational model (AlphaFold) to validate the predictions of another (ESM1b).

Transforming growth factor beta-3 (*TGFB3*) provides another example of isoform-sensitive variants. This proprotein is cleaved into two chains, LAP and TGFβ-3, that form a functional dimer. However, an alternative truncated isoform lacks the TGFβ-3 chain. ESM1b predicts many variants in the LAP chain as neutral only in the context of the truncated isoform, despite being over 200 residues away from the absent TGFβ-3 chain. While distant along the one-dimensional sequence, structure prediction from AlphaFold^44^ suggests close contact between these regions in 3D space (Fig. 5d).

### ESM1b can predict the effects of multiresidue variants

**Fig. 6:**
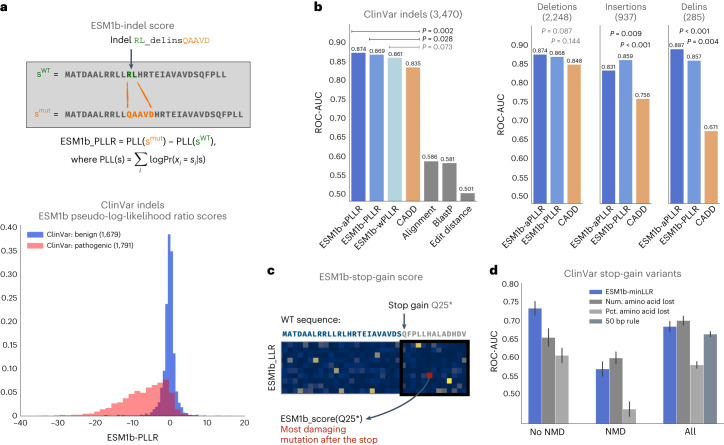
ESM1b effect predictions generalize to any coding variant. **a**, Top: functional effect scores are assigned to in-frame indels by invoking ESM1b on both the WT and mutated protein sequence and calculating the PLLR between them. Bottom: the distribution of ESM1b effect scores over 1,679 benign and 1,791 pathogenic in-frame indels from ClinVar. **b**, Comparison between three versions of ESM1b-based effect scores, CADD (a supervised VEP method) and three baseline models as classifiers of pathogenic versus benign in-frame indels (over the same set of variants as in **a**). One-tailed *P* values are shown for the differences between the performance of CADD and the ESM1b-based effect scores (Methods). Right: partitioning of the 3,470 in-frame indels into deletions, insertions and deletion–insertion combinations (delins). **c**, Functional effect scores are also assigned to stop-gain variants, defined as the LLR score assigned to the missense variant predicted to be the most deleterious among all possible missense variants in the lost region of the protein. Illustrated example: substitution of a glutamine into a stop codon at position 25. **d**, Assessment of ESM1b and three baseline models as classifiers of pathogenic versus benign stop-gain variants, over variants expected to either (1) not undergo NMD (3,672 pathogenic and 147 benign variants), (2) undergo NMD (32,362 pathogenic and 198 benign variants) or (3) all stop-gain variants (36,034 pathogenic and 345 benign variants). Error bars correspond to s.d. of the ROC-AUC scores centered around the mean (estimated by bootstrapping).

Unlike most VEP methods, protein language models can assess any amino acid sequence and, therefore, be leveraged to predict the effects of any coding mutation, including in-frame indels and stop gains. We use the term ‘indels’ to include insertions, deletions and deletion–insertion (delins) combinations. We defined the effect score of an in-frame indel to be the pseudo-log-likelihood ratio (PLLR) between the mutated and WT amino acid sequences, where the pseudo-log-likelihoods were estimated with ESM1b (Fig. 6a). Pathogenic indels, like missense variants, exhibit lower effect scores than benign indels (Fig. 6a).

**Extended Data Fig. 3:**
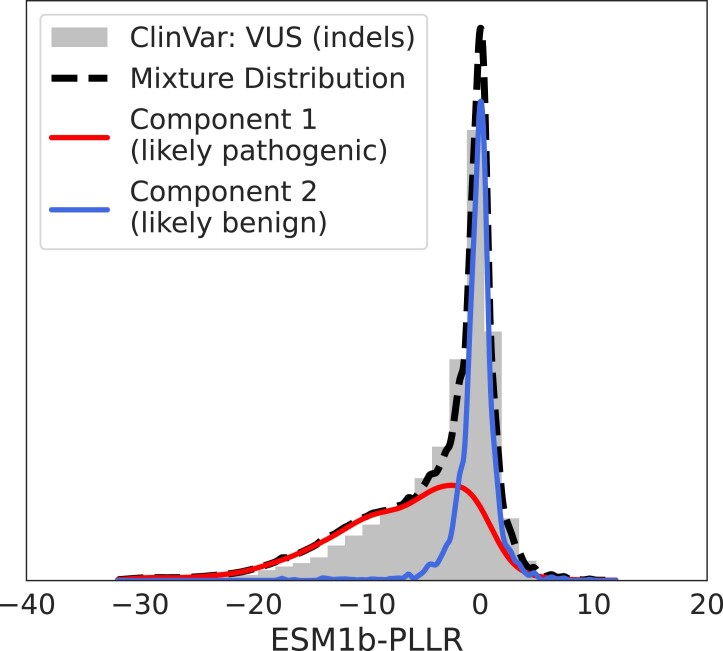
Estimating the pathogenicity rate among indel variants of uncertain significance. In gray: the distribution of ESM1b PLLR effect scores across indels in ClinVar annotated as variants of uncertain significance (VUS). We estimated the fraction of pathogenic and benign variants among these VUS indels by decomposing the VUS distribution of effect scores as a mixture of the distributions over pathogenic and benign variants (Fig. 6a) approximated by kernel density estimation. Red and blue curves: the mixture components of pathogenic and benign effect scores, respectively. Black dashed curve: the sum of the pathogenic (red) and benign (blue) components as an estimate of the empirical distribution of VUS (gray).

We compared ESM1b to other models as a classifier of pathogenic versus benign in-frame indels (Fig. 6b). We considered the following three variations of ESM1b PLLR scores: (1) vanilla PLLR, (2) weighted PLLR (accounting for indel size) and (3) absolute-valued PLLR, which considers functional changes as damaging whether they increase or decrease likelihood (Methods). The absolute-value PLLR marginally outperforms (ROC-AUC = 0.874) the vanilla (0.869) and weighted PLLR (0.861). All variations of ESM1b PLLR scores outperform CADD (0.835) which, unlike most VEP methods supporting indels, was not directly trained on ClinVar and could therefore be evaluated. The performance gap is especially significant for delins variants (ESM1b = 0.887, CADD = 0.671). Both ESM1b and CADD outperformed the following three baseline models: (1) edit distance (0.501), (2) pairwise sequence alignment (0.586) and (3) BlastP (0.581). We also computed ESM1b effect scores for all in-frame indel VUS in ClinVar and approximated this distribution as a mixture of the pathogenic and benign distributions (Extended Data Fig. 3), estimating that 52% of these indels are pathogenic (compared to 42% pathogenicity rate estimated for missense VUS).

Stop-gain variant effects can be predicted from the ESM1b scores for missense variants, by assigning each stop-gain an effect score determined by the lowest (that is most damaging) LLR score across all possible missense variants in the lost region following the new stop codon (Fig. 6c). Notably, ESM1b is a protein language model trained to assess protein sequence variations, while the effects of stop-gains are often at the transcript level through nonsense-mediated decay (NMD). Indeed, ESM1b is a good classifier for variants not resulting in NMD according to the 50 bp rule^45^ (ROC-AUC = 0.734) but performs poorly (0.565) over variants expected to cause NMD (Fig. 6d). Over the set of non-NMD variants, ESM1b substantially outperforms two baseline models scoring stop-gains based on the total number of residues lost (0.649) or their fraction of the WT protein length (0.599).

## Discussion

A comprehensive evaluation shows that ESM1b outperforms other state-of-the-art VEP methods at distinguishing pathogenic from benign variants across ClinVar and HGMD/gnomAD, and at predicting effects reported by DMS assays. As a protein language model that does not explicitly rely on homology, ESM1b offers several additional advantages for VEP. As an unsupervised method, ESM1b poses no risk of information leakage from the training to the test sets in clinical (for example, ClinVar and HGMD) or population genetics (for example, gnomAD) datasets, allowing accurate and unbiased evaluation. Prediction with ESM1b is much simpler and faster than with homology-based methods because only a single input sequence is required once a universal model has been trained. Notably, protein language models can provide predictions for every possible amino acid sequence and are applicable to all coding variants. In this work, the generalizability of ESM1b has been demonstrated for (1) variants outside MSA coverage, (2) variants with different effects on alternative protein isoforms, (3) in-frame indels and (4) stop-gain variants.

While homology-based VEP methods like EVE have a strong track record^4^, many important protein domains and variants are outside MSA coverage. Including regions with more distant homologs increases coverage but reduces MSA quality and method performance. Protein language models, on the other hand, are not directly affected by this tradeoff, as they are trained over all available sequences. Some recent strategies have integrated protein language models with homology-based methods, building on the complementary strengths of these two approaches and yielding promising prediction accuracy^46,47^.

Our workflow is unique in its ability to predict variant effects across alternative isoforms, unlike existing methods that can only determine whether a variant is included in an expressed isoform^48^ but not predict its unique effect in the context of that isoform. We highlighted 3,477 ClinVar missense variants with variable predicted effects between isoforms, present in many disease-causing genes including *BRCA1*, *IRF6* and *TGFB3*. Across the genome, ~1.8 million variants in ~9,000 genes were predicted to be isoform sensitive. While these numbers depend on definition thresholds, isoform-sensitive effects are clearly abundant. These variants tend to occur near splice sites and within genes containing splicing-disrupted domains, suggesting local effects, but some splicing events are predicted to influence much larger or distant protein regions. By combining isoform-specific effect predictions with isoform expression data (for example, from GTEx^49^), one could potentially trace the tissue affected by pathogenic variants.

Other concurrent works exploring ESM models for VEP over clinical and DMS data have obtained results largely consistent with ours, establishing protein language models as leading methods for this task^50,51^. By addressing the protein length limitation, our framework allows genome-wide predictions for all coding variants. Consequently, we compiled a complete catalog of all possible missense variant effects in the human genome (https://huggingface.co/spaces/ntranoslab/esm_variants). We further extended ESM1b to predict the effects of multiresidue variants, demonstrating good performance over in-frame indels (including deletion–insertion combinations) and stop-gains. While numerous VEP methods target missense variants, fewer can score more complex amino acid changes, with most trained on clinical databases like ClinVar.

Our framework comes with some limitations. Unlike VEP methods that use genomic features to assess variant effects at the DNA or transcript level, protein language models consider only amino acid changes. This limitation is demonstrated by the poor performance of ESM1b over variants leading to NMD. Similarly, ESM1b is not expected to detect variant effects on splicing^52^, but as shown, it can uncover isoform-specific effects at the protein level. Another limitation of the current framework is the lack of an explicit confidence metric for individual predictions, a feature offered by some VEP methods for quality control. Notably, this is not an inherent limitation of ESM1b or other protein language models, and future research will likely yield algorithms for quantifying prediction uncertainty. Finally, for the ~12% of human proteins too long for ESM1b to process as a single sequence, we employed a sliding window approach (Methods), which we expect to fail at detecting extremely distant interactions, specifically between residues separated by more than 1,022 amino acids.

We anticipate that our framework and public resource will be useful for a broad range of human genetics tasks. For diagnosing Mendelian diseases, integrating ESM1b effect scores with other information could help resolve the ambiguity of VUS. This remains a pressing need given the high prevalence of VUS in clinical sequencing^10^, which leaves many patients without a clear diagnosis^2,53–55^. For genetic association studies, using effect scores as priors could improve the power of variant burden tests and statistical fine-mapping^1^. For protein engineering, it has been shown that ESM1b effect scores can nominate gain-of-function variants with therapeutic benefits^56^. Lastly, using protein language models for VEP can provide insights into protein function, such as discerning functional differences between alternative isoforms or identifying protein domains and other functional units.

Over the past decades, computational VEP methods have dramatically improved^4^. Given the results presented in this work, and in line with the performance of language models in protein research^19,20,25,57^ and general machine learning^58,59^, protein language modeling stands out as one of the most promising approaches to determine the clinical and biological consequences of genetic variants. It has been shown that as language models scale in the number of parameters and training data, they tend to substantially improve^19,58^ (although this may not always be straightforward^60^). We expect that the trend of larger and better protein language models will continue to benefit and improve VEP.

## Methods

This study did not require any ethical approval.

### ESM1b

**Extended Data Fig. 4:**
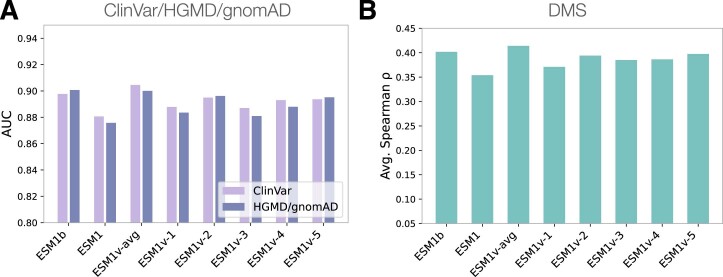
Evaluation and comparison of different ESM models. Tested ESM models: ESM1b, ESM1, the five ESM1v models, and an assembly of the five ESM1v models into a single model averaging the LLR scores obtained by the 5 models (ESM1v-avg). (**a**) Performance of the different ESM models on the clinical benchmarks (ClinVar and HGMD/gnomAD). Each model was evaluated as a binary classifier of pathogenic vs. benign missense variants over the two benchmarks using the global ROC-AUC metric. Only proteins smaller than 1,022aa were considered in this evaluation (thereby avoiding the sliding window approach). (**b**) Performance of the ESM models on the DMS benchmark.

In this study, we have leveraged and expanded the use of ESM1b, a protein language model developed by MetaAI^20^. The code and pretrained parameters for ESM1b (and other ESM models) were taken from the model’s official GitHub repository at https://github.com/facebookresearch/esm. Throughout this work, we used the esm1b_t33_650M_UR50S model (downloaded from https://dl.fbaipublicfiles.com/fair-esm/models/esm1b_t33_650M_UR50S.pt). Other ESM models, which are subtle variations of ESM1b, also exist and have been suggested specifically for the task of VEP (for example, ESM1v)^25^. Comparison of all ESM models, including ESM1b, ESM1 and the five ESM1v models, indicates that ESM1b is the best-performing ESM model over the three benchmarks used in this work, while an ensemble ESM1v model averaging the predictions of the five individual ESM1v models slightly outperforms ESM1b (Extended Data Fig. 4). In this work, we sought to explore the potential of a protein language model as a VEP method and therefore focused on a nonensemble model (ESM1b).

### Missense effect scores

ESM1b can compute the LLR scores for all possible missense mutations in a protein through a single pass of the neural network. With the WT amino acid sequence as input, ESM1b outputs the log-likelihood of each of the 20 standard amino acids (including the WT amino acid) at each position of the protein sequence. The LLR score of each mutation is the difference between the log-likelihood of the missense and WT amino acids at that position (Fig. 1b). Proteins longer than 1,022 amino acids are tiled through the sliding window approach described in the ‘Handling long sequences’ section below.

### Handling long sequences

**Extended Data Fig. 5:**
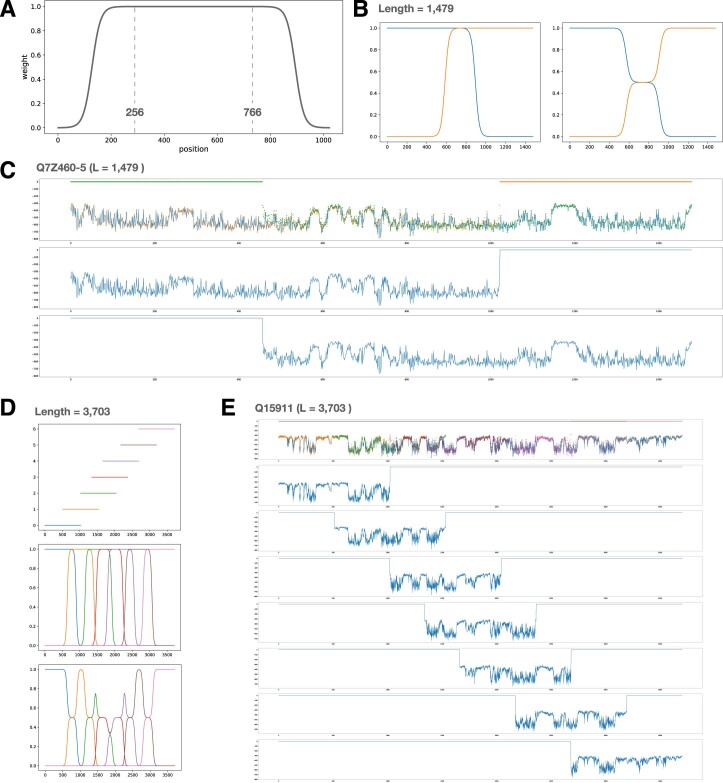
The sliding-window approach to tile long protein sequences with ESM1b. (**a**) The variant weights over each window’s coordinates (*1* ≤ *i* ≤ *1022*), defined by the function: *w(i)* = *1 / (1 + exp(-(i-128)/16)* for *1* ≤ *i* < *256*, *w(i)* = *1* for *256* ≤ *i* < *1022-256*, and *w(i)* = *1/(1 + exp((i-1022* + *128)/16)* for *1022-256* ≤ *i* ≤ *1022*. (**b**) An example tiling of a protein sequence of length 1,479aa. Left: raw window weights (as in (a)). Right: normalized weights (summing up to 1 at each protein position). (**c**) Example of how a specific protein isoform (UniProt ID Q7Z460*-*5) is tiled. Top panel: ESM1b effect scores over the left window (*1* ≤ *i* ≤ *1022*; orange), the right window (*458* ≤ *i* ≤ *1479*; green), and the final weighted average throughout the entire protein’s length (blue). Middle: ESM1b effect scores over the left window. Bottom: ESM1b effect scores over the right window. (**d**) An example tiling of a larger protein sequence of length 3,703aa, as in (b). Top: the locations of the 7 windows used to tile the sequence. Middle: raw window weights. Bottom: normalized weights. (**e**) Example of how a specific protein (UniProt ID Q15911) is tiled, as in (c). As shown in the two examples, the effect scores tend to be consistent across different windows (with edge effects sometimes being more pronounced).

ESM1b, using learned positional embeddings and self-attention (which grows quadratically in memory and compute), is limited to sequence lengths of up to 1,022 amino acids^20^. However, ~12% of human proteins in UniProt exceed this length^18^. To overcome this limitation, we employed a sliding window approach, subdividing longer sequences into overlapping 1,022 amino acid windows with at least 511 amino acid overlap (Extended Data Fig. 5). Each protein sequence was tiled by iteratively generating 1,022 amino acid window from both ends of the sequence such that consecutive windows had exactly 511 amino acid overlap until windows from both ends met at the center. If the overlap between the central windows was less than 511 amino acids, an additional 1,022 amino acid window was added at the center. The window subsequences were provided as inputs for ESM1b to compute the LLR scores for all missense variants (each variant with respect to all the windows containing it). With most residues covered by multiple overlapping windows (up to three windows, by construction), final variant effect scores were determined by a weighted average approach. To mitigate potential edge effects, weights near window edges were constructed with a sigmoid function (Extended Data Fig. 5a). A variant’s final effect score was calculated by (*w*(*i*1) × *s*1+*…*+*w*(*ik*) × *sk*) / (*w*(*i*1)+*…*+*w*(*ik*)), where *s*1*,…,sk* are the effect scores of the variant in the context of each of the *k* windows containing it (1 ≤ *k* ≤ 3), *i*1*,…,ik* are the variant’s positions in these windows, and *w* is the window weight function (Extended Data Fig. 5b–e).

**Extended Data Fig. 6:**
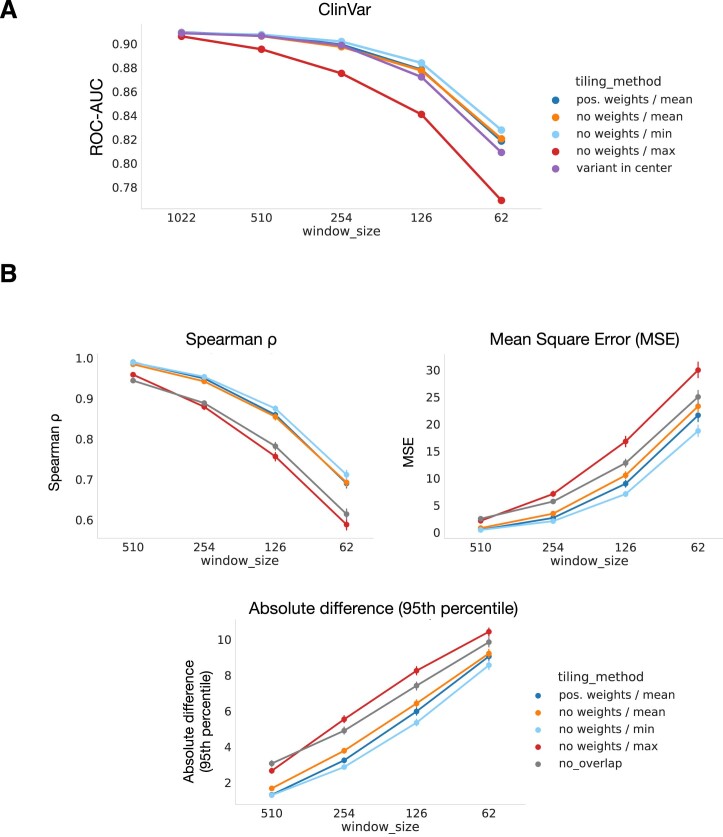
Evaluation of different sliding window approaches and window sizes. (**a**) Evaluation as binary classifiers of variant pathogenicity over the ClinVar dataset (global ROC-AUC metric). (**b**) Evaluation over short proteins (640 to 900aa), by comparing the scores obtained from processing the entire sequences through a single window vs. multiple windows. Three metrics are considered for comparing the scores: Spearman’s correlation (left), mean square error (center) or 95th percentile of absolute difference (right). Comparison was performed over 500 randomly chosen proteins of length 640 to 900aa. To accommodate different window sizes with the weighted-average approach, we rescaled the range of the sigmoid function (described in Extended Data Fig. 5) in proportion to the window size. Points along the curves correspond to the mean metric values across the 500 proteins; error bars correspond to 95% confidence intervals for the means.

We also considered other methods for tiling long sequences and aggregating effect scores across the 1–3 windows covering each variant. Besides the described weighted average, we tested (1) simple average (that is, without weights), (2) minimum (that is, the most damaging effect score), (3) maximum (that is, least damaging) and (4) placing the variant at the center of a single window. We compared the approaches in two complementary ways. First, we evaluated the five tiling approaches over the ClinVar benchmark with varying window sizes (Extended Data Fig. 6a), finding, as expected, that performance improves with window size. At a window size of 1,022 amino acids (the maximum supported by ESM1b), no approach outperformed the weighted average. Notably, placing each variant at the center of a single window is too inefficient for a genome-wide analysis as it processes each variant individually, whereas sliding window approaches invoke ESM1b once to process all the mutations in each window. As a second comparison, we quantified the error induced by using multiple windows as opposed to a single window (over short enough sequences that fit in one window). Once again, none of the alternative approaches is superior at the maximum window sizes (Extended Data Fig. 6b). Due to the compute burden, we omitted the variant-at-the-center approach in this comparison, considering instead a sliding window approach without overlap between consecutive windows.

### Generalized effect scores for indels and stop-gain variants

Unlike missense effect scores, computing generalized effect scores for in-frame indels requires the neural network to be invoked separately on each mutated sequence. The pseudo-log-likelihood of a sequence *s* = *s*_1_,…, *s*_*L*_ is calculated as ${\rm{PLL}}(s)={\sum }_{i=1}^{L}\log {\rm{Pr}}({x}_{i}={s}_{i}{|s})$, where *L* is the sequence length, *s*_*i*_ is the amino acid at position *i*, and log Pr(*x*_*i*_ = *s*_*i*_|*s*) is the log-likelihood predicted by ESM1b for observing the input amino acid *s*_*i*_ at position *i* given the entire input sequence *s*. In this framing, the output of ESM1b is considered a sequence of random variables *x* = *x*_1_,…,*x*_*L*_, where *x*_*i*_ expresses the probabilities of observing each of the 20 standard amino acids at position *i*. The effect score of an in-frame indel is the PLLR between the mutated and WT sequences: PLL(*s*^mut^) − PLL(*s*^WT^) (Fig. 6a).

Given the protein length limit of ESM1b, if either the WT or mutated sequences exceed 1,022 amino acids, PLLR is calculated using subsequences that satisfy this constraint. These subsequences include the region deleted and/or inserted by the indel together with unaffected regions before and after the indel (which are included as context for both the WT and mutated sequences). Before the indel, we include a segment of 511 residues (or as many as there are). After the indel, we include the number of residues that would complete the overall length to 1,022 amino acids, considering the longer between the WT or mutated sequence. The PLLs for the mutated and WT sequences are then calculated with respect to that window.

We refer to the PLLR score described above as ‘vanilla’ PLLR, while also considering the following two minor variations: (1) weighted PLLR and (2) absolute-valued PLLR (Fig. 6b). The weighted PLLR aims to account for a potential bias when the WT and mutated sequences have different lengths. Because LLR subtracts the sum of log-likelihoods across WT positions from that of the mutated sequence, there is a concern for subtracting incomparable values if the WT sequence length *L*_WT_ is too different from the mutated sequence length *L*_mut_. The weighted PLLR attempts to correct for that by replacing the vanilla subtraction PLL(*s*^mut^) − PLL(s^WT^) with $\frac{1}{{L}_{{\rm{mut}}}}{\rm{PLL}}({s}^{{\rm{mut}}})-\frac{1}{{L}_{{\rm{WT}}}}{\rm{PLL}}({s}^{{\rm{WT}}})$. The fact that the weighted PLLR does not outperform the vanilla PLLR (Fig. 6b) suggests that PLL scores predicted by ESM1b are overall well-calibrated likelihood estimates for sequences of varying lengths. The absolute-valued PLLR replaces the vanilla subtraction with |PLL(*s*^mut^) − PLL(*s*^WT^)|. The rationale for this transformation is to also consider variants that dramatically increase the overall likelihood of a protein as potentially pathogenic. For example, a gain-of-function mutation may appear more likely from an evolutionary perspective, yet such mutations are often pathogenic.

To score stop-gain variants, we initially compute missense LLR scores for the entire protein sequence (invoking the sliding window approach if needed). The effect score of a stop-gain variant is then chosen to be the lowest LLR score (that is predicted most damaging) among all possible missense mutations in the lost region (Fig. 6c). The rationale is to assess how important the lost region at the end of the protein is to its function, and assign lower scores the more functionally important it is. As demonstrated by the analysis of protein domains (Figs. 1d and 3d), functionally important protein regions contain missense mutations with lower ESM1b scores.

### AUC metrics for pathogenicity classification

To compare the performance of ESM1b and other VEP methods as variant pathogenicity classifiers, we primarily used ROC-AUC (Fig. 2b,e–h), the standard evaluation metric for binary classifiers^61^. In addition to ROC-AUC, which considers the tradeoff between the true- and false-positive rates (Extended Data Fig. 1a), we also used a balanced version of the PRC-AUC metric, which considers the tradeoff between precision and recall (Fig. 2e,f). Unlike ROC-AUC, PRC-AUC is generally sensitive to label imbalance (that is, an uneven split of pathogenic/benign variants) in the evaluation dataset. To balance this metric, we randomly downsampled each dataset into an equal number of pathogenic and benign variants (80% of the variants in the minority class) and calculated the PRC-AUC over the balanced dataset. To obtain accurate scores, we repeated downsampling 100 times and calculated the average of the resulting PRC-AUC scores.

We treated the entire set of pathogenic and benign variants (from ClinVar^10^ or HGMD/gnomAD^9,26^) as a single genome-wide classification task to calculate a global ROC-AUC. This is somewhat different from the gene-average ROC-AUC reported in the publication introducing EVE^4^. Under the gene-average approach, each gene was evaluated separately, yielding a gene-specific ROC-AUC for the 1,654 human genes with at least one annotated ClinVar variant per class (pathogenic/benign). Averaging across these genes gave the gene-average ROC-AUC. ESM1b is consistently superior to all other methods according to the global ROC-AUC (Fig. 2b,e–h), while EVE is somewhat superior according to the gene-average ROC-AUC over this subset of genes (Extended Data Fig. 1b). This suggests that ESM1b provides scores that are more consistent and comparable across different genes, which may be attributed to EVE being an assembly of multiple gene-specific models, whereas ESM1b is a universal model trained over all known protein sequences. We argue that global ROC-AUC is usually more informative than gene-average ROC-AUC for VEP, as diagnosing genetic diseases often involves comparing variants across multiple genes, requiring well-calibrated scores.

In Fig. 6d, we estimated uncertainty for the ROC-AUC metrics through bootstrapping. In each bootstrapping iteration, we randomly sampled 140 pathogenic and 140 benign variants from each of the three groups of stop-gain variants (3,672 pathogenic and 147 benign variants not expected to lead to NMD, 32,441 pathogenic and 198 benign variants expected to lead to NMD, and 36,113 pathogenic and 345 benign variants overall). Following 20 iterations, we calculated the mean ROC-AUC and s.d. (presented as error bars in Fig. 6d) for each condition.

### Other VEP methods

Other than ESM1b and EVE, we evaluated 44 other VEP methods (Figs. 2 and 3). Predicted effect scores for most VEP methods were taken from dbNSFP^33^. We used the dbnsfp4.3a.zip file from the dbNSFP website (http://database.liulab.science/dbNSFP). We excluded LINSIGHT (which had too few variants for reliable evaluation) and three versions of fitCons based on the H1-hESC, HUVEC and GM12878 cell lines (which showed near random performance on ClinVar and HGMD/gnomAD). We further included two other recent state-of-the-art methods not reported in dbNSFP—VARITY (consisting of the following two versions: VARITY_R and VARITY_ER)^62^ and MTBAN^63^.

Of the 46 VEP methods, 19 meet the criteria for evaluation on clinical benchmarks for missense variants (ClinVar and HGMD/gnomAD), having avoided training on clinical databases, using features from other methods trained on such data, or using allele frequency (Supplementary Table 2). DMS assays generally avoid this data leakage issue, hence we compared all 46 methods on the DMS benchmark. To allow unbiased evaluation of VARITY on the DMS benchmark, we excluded the variants included in its training (provided in the method’s GitHub repository at https://github.com/joewuca/varity). Both VARITY and MTBAN were excluded from the comparison over the set of DMS variants available for all methods (Fig. 3a), to prevent a significant reduction in the number of variants and genes. Specifically, VARITY was trained on five genes (*BRCA1*, *CBS*, *MSH2*, *MTHR* and *PTEN*) and MTBAN misses three other genes (*A4*, *SYUA* and *YAP1*) of the 11 genes in that comparison. Both methods were still included in the direct comparison against ESM1b (Fig. 3c).

### Baseline scores of indel and stop-gain variant effects

While numerous VEP methods predict missense variant effects (46 evaluated here; Figs. 2 and 3), few handle indel and stop-gain variants. The vast majority of these have been trained on clinical databases like ClinVar, leading to circularity issues when evaluating them on the same benchmarks. Therefore, we compared ESM1b to only one other VEP method (CADD) over the ClinVar benchmark of in-frame indels (Fig. 6b) and none over stop-gain variants (Fig. 6d). To provide context for the performance of ESM1b on these benchmarks, we considered several basic scoring algorithms that we consider reasonable baselines.

For in-frame indels, we considered baseline scores based on the followings: (1) edit distance, (2) pairwise alignment and (3) BlastP. The Levenshtein edit distance determines the minimal number of single-amino acid operations (insertions, deletions or substitutions) needed to transform the WT into the mutated sequence. The pairwise alignment score reflects the overall similarity between the WT and mutated sequence after they are aligned (match score = 2, mismatch score = −1)^64^. BlastP uses the same alignment algorithm with a scoring system that also takes into account the different amino acid propensities (with BLOSUM62 (ref. ^65^)) and panelizes gaps. All three scores share the same premise that the more dissimilar the WT and mutated sequences are, the more likely the indel to be damaging.

For stop-gain variants, we considered the following baseline scores: (1) the number of residues lost, (2) the percentage of residues lost (relative to the WT sequence length) and (3) the 50 bp rule. Considering the number or percentage of lost residues shares the premise that larger lost regions are more likely to be damaging. The 50 bp rule asserts that a transcript is likely to undergo NMD only if a stop codon is introduced more than 50 base pairs upstream of the last exon junction within its coding region^45^. We applied the 50 bp rule based on exon annotations in the human genome (Supplementary Methods). Unlike the other baselines that provide continuous scores, the 50 bp rule provides binary labels.

### Testing for significant performance differences

When comparing the performance of ESM1b to that of other VEP methods across benchmarks (Figs. 2b,g,h, 3c and 6b), statistical significance was determined through permutation tests. In each iteration, we shuffled the effect scores assigned by each method between the benchmark’s variants, and recalculated the output metric (AUC score or Spearman’s correlation) for ESM1b and the compared method. The empirical one-tailed *P* value was the fraction of 2,000 iterations where the difference in output metric was as extreme as that with the actual, nonpermuted effect scores. If no permutations gave a difference as large as the one measured for the true effect scores, we reported *P* < 0.001.

### DMS

We evaluated 46 VEP methods, including ESM1b and EVE, on a DMS benchmark spanning 28 assays across 15 genes. We used the same set of human genes as in ref. ^4^ (excluding Rhodopsin^66^ due to unavailable public data), and added three other genes from MaveDB^11^. We downloaded all accessible experimental data for these assays (Supplementary Table 1).

Throughout our evaluation, we used the raw experimental scores without any further processing for all DMS, except for CALM1, TPK1, RASH, TADBP and the abundance assay of SYUA. For these assays, we transformed the scores by *x* → *|x* *–* *x*_WT_|, where *x*_WT_ denotes the assay-wide value measured for WT. The motivation for this transformation is that variants scoring higher than WT are typically seen as deleterious in these assays (see discussions in refs. ^67,68^). For SYUA, as lower abundance variants are less toxic, the abundance scores were transformed the same way to better reflect fitness (Supplementary Fig. 2 in ref. ^69^). This preprocessing noticeably improved the performance of all VEP methods on these assays.

For each assay, we calculated Spearman’s rank correlation between the assay scores and each VEP method’s predictions. We then averaged these correlation coefficients per gene, which may encompass multiple assays (Fig. 3b and Extended Data Figs. 1c and 2). Finally, we averaged the per-gene averages (Fig. 3a,c).

### Statistics and reproducibility

All data used in this work is within the public domain (except HGMD, which requires access request). The full benchmark datasets and Python code for our ESM1b-based workflow are available on our GitHub repository (Data availability and Code availability statements). For details on our statistical analysis, see the subsection ‘Testing for significant performance differences’. No statistical method was used to predetermine the sample size.

### Reporting summary

Further information on research design is available in the Nature Portfolio Reporting Summary linked to this article.

## Online content

Any methods, additional references, Nature Portfolio reporting summaries, source data, extended data, supplementary information, acknowledgements, peer review information; details of author contributions and competing interests; and statements of data and code availability are available at 10.1038/s41588-023-01465-0.

### Supplementary information

Supplementary Information Supplementary Table 1 and Methods.

Reporting Summary

Supplementary Table 2 Full benchmark evaluations of ESM1b and all other methods.

## Data Availability

All data used in this study are already within the public domain, with the exception of the HGMD dataset (https://www.hgmd.cf.ac.uk/ac/index.php), which is a private resource owned by the Institute of Medical Genetics in Cardiff University (requests to access this database should be directed to its curators). ClinVar labels for missense, indel and stop-gain variants were downloaded directly from ClinVar’s website (https://ftp.ncbi.nlm.nih.gov/pub/clinvar/tab_delimited/variant_summary.txt.gz). A specific ClinVar benchmark with EVE scores was downloaded from the EVE portal (https://evemodel.org/). Details on how the datasets and benchmarks were processed are available in Supplementary Methods. Predicted effect scores for most VEP methods were downloaded from dbNSFP (http://database.liulab.science/dbNSFP). Details on the remaining VEP methods are available in the ‘Other VEP methods’ section in Methods. We also provide all processed benchmarks, with effect scores from all VEP methods compared in this work, on our GitHub repository (link below). All benchmark results are in Supplementary Table 2. The complete catalog of variant effect scores predicted by ESM1b for all possible missense variants affecting curated protein isoforms in the human genome can be browsed and downloaded through our web portal at https://huggingface.co/spaces/ntranoslab/esm_variants.

## Code availability

Code for calculating variant effect scores with our framework and processed data files are available on our GitHub repository (https://github.com/ntranoslab/esm-variants). All the code and data for producing the analysis, figures and results presented in this study are available on Zenodo^70^.
