# Supplementary material for: Genome-wide prediction of disease variant effects with a deep protein language model
Source: Nat Genet. 2023 Aug 10;55(9):1512–22. doi: 10.1038/s41588-023-01465-0 (PMC10484790; doi:10.1038/s41588-023-01465-0)
Supplement: Supplementary file 1 — Supplementary Table 1 and Methods. [file 41588_2023_1465_MOESM1_ESM.pdf]

nature genetics

Article

https://doi.org/10.1038/s41588-023-01465-0

Genome-wide prediction of disease variant effects with a deep protein language model

In the format provided by the authors and unedited

Supplementary Table 1: Deep mutational scans used for model evaluation

Human gene	Assays	Source	Description
ADRB2	Activity in response to four concentrations of an agonist (Control, 150nM, 625nM, 5uM)	Jones 2020 (doi.org/10.7554/eLife.54895), Supplementary file 2	RNA-seq of barcoded reporter gene stimulated by cAMP in HEK293-derived cell line.
A4	Nucleation score	Seuma 2020 (doi.org/10.7554/eLife.63364), Supplementary file 4	Growth-based selection due to A4 limiting aggregation of the sup35 prion in yeast.
BRCA1	Function score	Findlay 2018 (doi.org/10.1038/s41586-018-0461-z), Supplementary table 1	Cas9/gRNA construct transfected into HAP1 cell line followed by targeted DNA sequencing to quantify abundance of each mutant.
CALM1	Fitness score	Weile 2017 (doi.org/10.15252/msb.20177908), Dataset EV1	Abundance of yeast strain *ubc9-ts* carrying mutant (determined by sequencing); DMS-TileSeq.
MSH2	LoF score	Jia 2020 (doi.org/10.1016/j.ajhg.2020.12.003), Supplementary Data 1	Mismatch repair dysfunction and deep sequencing to identify the surviving MSH2 variants.
P53	Activity in three experimental conditions (WT_Nutlin-3, NULL_Nutlin-3, NULL_Etoposide)	Giacomelli 2018 (doi.org/10.1038/s41588-018-0204-y) Supplementary Table 3	Mutagenesis by Integrated TilEs (MITE) in A549 human lung carcinoma cell populations followed by pooled positive selection screens in nutlin-3 or etoposide.

PTEN	Fitness score	Mighell 2018 (doi.org/10.1016/j.ajhg.2018.03.018), Table S2	Humanized yeast model to assess the phosphatase activity of PTEN variants.
RASH	Growth assay in three experimental conditions (Attenuated, Regulated, Unregulated)	Bandaru 2017 (doi.org/10.7554/eLife.27810), Supplementary file 1	Selection on antibiotic resistance due to resistance gene driven by Ras-Raf binding in yeast.
SYUA	Fitness score, Abundance score	Newberry 2020 (doi.org/10.1038/s41589-020-0480-6), Source data 2	Expression of α-synuclein in yeast slows growth in a dose-dependent manner that can be modified by point mutations.
TPK1	Fitness score	Weile 2017 (doi.org/10.15252/msb.20177908), Dataset EV1	Abundance of yeast strain *ubc9-ts* carrying mutant (determined by sequencing); DMS-TileSeq.
VKOR1	Abundance score, Activity score	Chiasson 2017 (doi.org/10.7554/eLife.58026), Source data 1	Variant Abundance by Massively Parallel sequencing (VAMP-seq) and activity reporter assay
YAP1	Binding affinity	Araya 2012 (mavedb.org/scoreset/urn:mavedb:00000002-a-2/)	Binding affinity between the human YAP65 (YAP1) WW domain and a peptide binding partner using phage display.
MTHR	Functional complementation in yeast at 12, 25,100 and 200 ug/ml folate in WT background	Weile 2021 (mavedb.org/urn:mavedb:00000049-a)	Abundance of yeast strain carrying mutant; DMS-TileSeq.
CBS	Growth assay in low (0, 1ng/ml) or high (400 ng/ml) concentrations of vitamin B6	Sun 2020 (https://www.mavedb.org/experiment/urn:mavedb:00000005-a/)	Abundance of yeast strain carrying mutant; DMS-TileSeq.

TADBP	Growth assay to measure toxicity	Bolognesi 2019 (https://www.mavedb.org/experiment/urn:mavedb:00000060-a/)	Abundance of yeast strain carrying mutant; DiMSum

Supplementary Methods

Protein isoforms

Our analysis of protein isoforms is based on 42,336 manually-reviewed protein isoform sequences taken from UniProt [18] (in February 2022). To get ClinVar labels across all annotated variants and map them to UniProt protein sequences, we downloaded the *variant_summary.txt.gz* file from ClinVar (on April 29, 2022). This dataset contained information about ~1.3M variants, including clinical significance and NM code (RefSeq mRNA record). From a separate dataset on ClinVar's website (*hgvs4variation.txt.gz*, downloaded on May 3, 2022), we obtained a mapping between the NM codes and NP codes (RefSeq protein records). We then matched those NP codes with UniProt IDs, using the same set of 42,336 protein isoforms from UniProt. Following this mapping, from NM code to NP code to UniProt ID, we obtained 386,033 missense variants with clinical significance contributing to 848,320 effects on 12,179 UniProt isoforms (~2.2 effects per variant on average) across 5,683 unique genes. These variants were used in the analysis of ClinVar over alternative isoforms (Fig. 4).

Clinical benchmarks of missense variants (ClinVar and HGMD/gnomAD)

For the benchmarks incorporating only the primary isoforms (Fig. 2, Extended Data Fig. 1A-B, Extended Data Fig. 4 and Extended Data Fig. 6A), we used 46,726 variants with high-quality ClinVar labels (i.e., at least 1 "Gold Stars") across ~3K disease genes downloaded from the EVE portal (https://evemodel.org/). Importantly, this dataset includes all ClinVar labels for these genes (including variants not covered by EVE).

Access to the full HGMD dataset (https://www.hgmd.cf.ac.uk/ac/index.php) was provided upon request (see Acknowledgements) [26]. Of the entire set of 331,912 HGMD variants, 172,461 were missense variants, of which 158,725 were mapped to a unique UniProt isoform, of which 98,086 were annotated as high-confidence disease-causing variants (DM).

To create a list of common missense variants, we used exomed-derived variants from the gnomAD database (version 2.1.1), by downloading the file *gnomad.exomes.r2.1.1.sites.vcf.bgz*. From this VCF file, we extracted 5,624,824 missense variants with a PASS filter, of which 4,876,367 were called in over 200,000 alleles (according to the AN value in the VCF file) and were considered high-quality variants. Of these, 26,359 variants (0.5%) had MAF > 0.01 and were considered common variants.

Altogether, we had 124,445 variants in the HGMD/gnomAD benchmark (98,086 pathogenic and 26,359 likely benign variants). Of these, 92,942 variants (70,714 pathogenic and 22,228 benign) affected the primary UniProt isoform and were used in this benchmark throughout this work.

Indels and stop gain ClinVar benchmarks

The set of all ClinVar variants was downloaded from ClinVar's FTP site on January 24, 2023 (https://ftp.ncbi.nlm.nih.gov/pub/clinvar/tab_delimited/variant_summary.txt.gz). Of the 1,596,737 variants in version GRCh38 of the human reference genome, we detected 3,511 high-quality (i.e., at least 1 "Gold Stars") in-frame indels. The protein sequence matching each variant was determined by the reported RefSeq identifier. The amino acid changes reported for 17 of the 3,511 indels didn't match the RefSeq protein sequence. Filtering out these 17 variants, and 24 additional variants which were duplicates or included non-standard amino-acids, the final indel benchmark included 1,679 benign and 1,791 pathogenic variants. Notably, 3% of these indels included insertions or deletions of more than 17 residues (50nt), which are often considered structural variation rather than indels.

We further detected 36,489 high-quality (i.e., at least 1 "Gold Stars") stop-gain variants. To determine whether the 50bp rule [45] applies for each of these variants, we recovered the coordinate of the last exon junction in the coding region of the affected gene based on the exon annotations in RefSeq. Of the 36,489 variants, 110 didn't have exon annotations and were filtered out. The remaining dataset included 36,034 pathogenic and 345 benign stop-gain variants.

EVE scores and MSA coverage

EVE scores for missense variants affecting the ~3K disease-associated genes analyzed by the model were downloaded from the official EVE portal (https://evemodel.org/). From the same portal, we also downloaded multiple sequence alignments (MSAs) for the same ~3K disease genes. For each MSA, the target (human) protein sequence is reported as a combination of uppercase and lowercase letters. The uppercase letters correspond to residues that are part of the MSA profile, whereas lowercase letters correspond to residues that are not. Accordingly, we defined the coverage of a human protein (reported in **Fig. 1C-D**) to be the fraction of the target sequence letters that are uppercase.
