# Supplementary material for: Genome-wide prediction of disease variant effects with a deep protein language model
Source: Nat Genet. 2023 Aug 10;55(9):1512–22. doi: 10.1038/s41588-023-01465-0 (PMC10484790; doi:10.1038/s41588-023-01465-0)
Supplement: Supplementary file 2 — Reporting Summary [file 41588_2023_1465_MOESM2_ESM.pdf]

nature portfolio

Corresponding author(s): Vasilis Ntranos, Jimmie Ye

Last updated by author(s): Apr 3, 2023

Reporting Summary

Nature Portfolio wishes to improve the reproducibility of the work that we publish. This form provides structure for consistency and transparency in reporting. For further information on Nature Portfolio policies, see our Editorial Policies and the Editorial Policy Checklist.

Please do not complete any field with "not applicable" or n/a. Refer to the help text for what text to use if an item is not relevant to your study.
For final submission: please carefully check your responses for accuracy; you will not be able to make changes later.

Statistics

For all statistical analyses, confirm that the following items are present in the figure legend, table legend, main text, or Methods section.

n/a	Confirmed	
☐	☒	The exact sample size (*n*) for each experimental group/condition, given as a discrete number and unit of measurement
☐	☒	A statement on whether measurements were taken from distinct samples or whether the same sample was measured repeatedly
☐	☒	The statistical test(s) used AND whether they are one- or two-sided<br>*Only common tests should be described solely by name; describe more complex techniques in the Methods section.*
☒	☐	A description of all covariates tested
☐	☒	A description of any assumptions or corrections, such as tests of normality and adjustment for multiple comparisons
☐	☒	A full description of the statistical parameters including central tendency (e.g. means) or other basic estimates (e.g. regression coefficient) AND variation (e.g. standard deviation) or associated estimates of uncertainty (e.g. confidence intervals)
☐	☒	For null hypothesis testing, the test statistic (e.g. *F*, *t*, *r*) with confidence intervals, effect sizes, degrees of freedom and *P* value noted<br>*Give P values as exact values whenever suitable.*
☒	☐	For Bayesian analysis, information on the choice of priors and Markov chain Monte Carlo settings
☒	☐	For hierarchical and complex designs, identification of the appropriate level for tests and full reporting of outcomes
☐	☒	Estimates of effect sizes (e.g. Cohen's *d*, Pearson's *r*), indicating how they were calculated

Our web collection on statistics for biologists contains articles on many of the points above.

Software and code

Policy information about availability of computer code

Data collection	The data collection pipeline (for the ClinVar, HGMD/gnomAD and DMS benchmars, and for the 42,336 manually-reviewed protein isoforms across the human genome) is described in the Supplementary Methods.
Data analysis	Code for calculating variant effect scores with our framework is available on our GitHub repository (https://github.com/ntranoslab/esm-variants).

For manuscripts utilizing custom algorithms or software that are central to the research but not yet described in published literature, software must be made available to editors and reviewers. We strongly encourage code deposition in a community repository (e.g. GitHub). See the Nature Portfolio guidelines for submitting code & software for further information.

Data

Policy information about availability of data

All manuscripts must include a data availability statement. This statement should provide the following information, where applicable:

- Accession codes, unique identifiers, or web links for publicly available datasets
- A description of any restrictions on data availability
- For clinical datasets or third party data, please ensure that the statement adheres to our policy

All data used in this work is already within the public domain, with the exception of the HGMD dataset (https://www.hgmd.cf.ac.uk/ac/index.php) which is a private resource owned by the Institute of Medical Genetics in Cardiff University (requests to access this database should be directed to its curators). ClinVar labels of

missense variants in all protein isoforms and of indels and stop-gains were downloaded directly from ClinVar's website (https://ftp.ncbi.nlm.nih.gov/pub/clinvar/tab_delimited/variant_summary.txt.gz). A specific ClinVar benchmark incorporating only the primary isoforms and EVE scores were downloaded from the EVE portal (https://evemodel.org/). Full details on how the datasets and benchmarks were processed are available in the Supplementary Methods. Predicted effect scores for most VEP methods were downloaded from dbNSFP (http://database.liulab.science/dbNSFP). Details on the remaining VEP methods are available in the "Other VEP methods" section. We also provide all the processed benchmarks, including the effect scores from all VEP methods compared in this work, on our GitHub repository (link below). All benchmark results presented in this work are available in Supplementary Table S2.

Human research participants

Policy information about studies involving human research participants and Sex and Gender in Research.

Reporting on sex and gender	n/a
Population characteristics	n/a
Recruitment	n/a
Ethics oversight	n/a

Note that full information on the approval of the study protocol must also be provided in the manuscript.

Field-specific reporting

Please select the one below that is the best fit for your research. If you are not sure, read the appropriate sections before making your selection.

☒ Life sciences ☐ Behavioural & social sciences ☐ Ecological, evolutionary & environmental sciences

Life sciences study design

All studies must disclose on these points even when the disclosure is negative.

Sample size	No statistical method was used to predetermine sample size.
Data exclusions	No data was excluded from the analysis.
Replication	Having relied on well-established databases (rather than conducting new experiments), the concept of "replication" is irrelevant to this work.
Randomization	Having relied on well-established databases (rather than conducting new experiments), the concept of "randomization" is irrelevant to this work.
Blinding	Having relied on well-established databases (rather than conducting new experiments), the concept of "blinding" is irrelevant to this work.

Reporting for specific materials, systems and methods

We require information from authors about some types of materials, experimental systems and methods used in many studies. Here, indicate whether each material, system or method listed is relevant to your study. If you are not sure if a list item applies to your research, read the appropriate section before selecting a response.

Materials & experimental systems

n/a	Involved in the study	
☒	☐	Antibodies
☒	☐	Eukaryotic cell lines
☒	☐	Palaeontology and archaeology
☒	☐	Animals and other organisms
☒	☐	Clinical data
☒	☐	Dual use research of concern

Methods

n/a	Involved in the study	
☒	☐	ChIP-seq
☒	☐	Flow cytometry
☒	☐	MRI-based neuroimaging

This checklist template is licensed under a Creative Commons Attribution 4.0 International License, which permits use, sharing, adaptation, distribution and reproduction in any medium or format, as long as you give appropriate credit to the original author(s) and the source, provide a link to the Creative Commons license, and indicate if changes were made. The images or other third party material in this article are included in the article's Creative Commons license, unless indicated otherwise in a credit line to the material. If material is not included in the article's Creative Commons license and your intended use is not permitted by statutory regulation or exceeds the permitted use, you will need to obtain permission directly from the copyright holder. To view a copy of this license, visit http://creativecommons.org/licenses/by/4.0/
